# Early cholestasis in neonatal lupus erythematosus

**DOI:** 10.4103/0256-4947.70569

**Published:** 2011

**Authors:** Mozhgan Shahian, Amir Khosravi, Mohammad-Hossein Anbardar

**Affiliations:** From the Division of Neonatology, Department of Pediatrics, Shiraz University of Medical Sciences, Shiraz, Iran

## Abstract

Neonatal lupus erythematosus is an immune-mediated disease caused by transplacental passage of maternal autoantibodies, primarily anti-Ro (SSA) and anti-La (SSB). The major clinical manifestations are congenital heart block, cutaneous lupus lesions, and hematologic problems. Hepatic, pulmonary, and neurological involvements are rare. We report a 5-day-old male neonate, born to a clinically asymptomatic mother, presenting with conjugated hyperbilirubinemia, cutaneous lupus lesions, congenital heart block, and thrombocytopenia. Both the neonate and his mother had high titers of antinuclear antibodies (1:640), anti-Ro (SSA), and anti-La (SSB) antibodies. The thrombocytopenia improved with prednisolone (2 mg/kg/day) for 14 days. The skin lupus rashes and bilirubin resolved 2 months later, and liver enzymes were completely normal by 6 months.

Neonatal lupus erythematosus (NLE) is a rare immune-mediated disease characterized by the transplacental passage from the mother to the fetus of autoantibodies, in particular SSA or SSB or both. The common clinical manifestations of NLE include cardiac disease, cutaneous lesions, and hematologic problems.^1^ Recently, it has become clear that hepatobiliary disease may also occur as a manifestation of NLE. Although biochemical evidence of liver disease is common in patients with systemic lupus erythematosus (SLE), clinical liver disease is uncommon.

## CASE

A 5-day-old male neonate presented with generalized jaundice. He had been born by vaginal delivery at term with a birth weight about 2100 g. The mother (gravida: 6, para: 6, living: 6) was healthy with no significant past medical history other than mild photosensitivity. The baby had no history of passing clay-colored stools or of fever and had not been given any drugs.

On admission, physical examination revealed pale conjunctiva, icteric sclera, generalized jaundice, a few erythematous lesions in the periorbital areas, and mild splenomegaly. He had an irregular pulse, with a heart rate of about 75 beats per minute. The electrocardiogram showed third-degree atrioventricular block; echocardiography showed a patent foramen ovale but no other abnormality. Hematological investigation revealed anemia (hemoglobin 9 g/dL) and thrombocytopenia (platelets 80 000/mm^3^). A blood smear showed erythrocyte hypochromia, anisocytosis, and poikilocytosis. The reticulocyte index, C-reactive protein, and erythrocyte sedimentation rate were normal. Liver function tests showed increased values, as follows: aspartate aminotransferase (AST), 760 U/L (normal:1-46 U/L); alanine aminotransferse (ALT), 187 U/L (normal: 1-49 U/L); alkaline phosphate, 2045 U/L (normal: 64-306 U/L); total bilirubin, 12.4 mg/dL (normal: 0.1-1.3 mg/dL); and direct bilirubin, 6.2 mg/dL (normal: 0.1-1.3 mg/dL). Prothrombin time and partial thromboplastin time were not prolonged. TORCH titers, viral hepatitis markers, and thyroid function tests were normal. For both mother and neonate, blood and urine cultures were negative. Tests for metabolic diseases, including galactosemia, tyrosinemia, and phenylketonuria, were negative.

Abdominal ultrasound revealed a normal-sized liver and gall bladder, no bile duct dilation, and no sludge in the biliary tree. The spleen was mildly enlarged but showed a normal echo pattern. Hepatobiliary scintigraphy showed decreased hepatic uptake, with no passage through the intrahepatic bile ducts.

At first, the cutaneous lesions consisted of a few nonscarring erythematous annular plaques in the periorbital areas. Over the next few days, they spread to the nasal bridge and the upper parts of the cheeks and had sharp and slightly hyperkeratotic borders.

Serologic studies of the infant and mother were positive for antinuclear antibodies (ANA; 1: 640), anti-Ro/SSA: >4 index (normal: <1 index) and anti-La/SSB antibodies: >4 index (normal: <1 index). Anti-ds DNA antibodies, anti-SM antibodies, anti-U RNP antibodies were not detected. More detailed studies in the mother revealed a low C3 level of 0.71 g/dL (normal: 0.9-1.87 g/dL), leukopenia (WBC: 2900 mm^3^), and anemia (hemoglobin: 10.6 mg/dL).

We diagnosed NLE. Earlier studies have reported beneficial effects of glucocorticoids on different manifestations of NLE such as thrombocytopenia and cholestasis, and we therefore prescribed prednisolone (2 mg/kg/day) for 2 weeks along with ursodeoxycholic acid. The parents were advised to avoid exposing the neonate to the sun and to use sunscreen agents and topical hydrocortisone creams. After the platelet count had returned to normal the patient was discharged and was then followed up in the outpatient clinic. Within 2 months both the jaundice and the skin rashes had resolved. At 6 months of age, liver function tests were normal. During the follow-up period, the patient had a normal heart rate and there was no evidence of heart failure.

## DISCUSSION

NLE results from maternal transfer of IgG autoantibodies between the 12th and 16th week of gestation.^2^ Ninety-eight percent of NLE babies have anti-Ro antibodies but only 1% to 2% of mothers with SSA/Ro antibodies have neonates with NLE, irrespective of whether the mothers are symptomatic or not. A considerable proportion of mothers of affected infants are asymptomatic (40% to 60%), while the remaining women have clear evidence of SLE, Sjögren syndrome, or of some undifferentiated connective tissue disease.

The clinical manifestations of NLE may include congenital heart block (CHB), cutaneous lesions, thrombocytopenia, pulmonary and neurologic disease, and hepatitis. Of these, CHB and cutaneous lesions are the most common, occurring in 54% and 37% of cases, respectively.^2^

The characteristic skin lesions of NLE have a predilection for the upper and lower eyelids, giving rise to a typical ‘owl-eye’ appearance in the majority of babies. The trunk and extremities are also commonly affected. These lesions may be erythematous, annular, raised or flat, and sometimes show fine scales. They develop within hours to several days after delivery and follow exposure to sunlight.^3^ The lesions typically disappear spontaneously, without the disappearance of the serum antibodies. However, a few cases of persistent telangiectasia, atrophy, or pigmentation have been reported.

The most common cardiac manifestation of neonatal lupus is CHB, which is most commonly diagnosed between 18 and 24 weeks of gestation and may be of first, second, or third degree. Established third-degree block appears to be irreversible. In some cases, cardiomyopathy occurs together with CHB. Most cases have been noted at birth, but delayed dilated cardiomyopathy has also been reported.^4^ Treatment of the heart block is not necessary unless the patient has heart failure.^5^ CHB carries a significant mortality (15% to 30%) and morbidity, with 50% to 70% of patients requiring permanent pacing.

In early studies, liver involvement in NLE was mentioned as a rare condition, but now it appears that about 10% to 24% of NLE cases have liver involvement. Three types of hepatobiliary disease have been described: 1) severe liver failure occurring during gestation or in the neonatal period; 2) conjugated hyperbilirubinemia with mild or no elevation of aminotransferases, occurring during the first weeks of life; and 3) mild elevation of aminotransferases occurring at 2 to 3 months of age.^6^ Various forms of hepatic pathology related to SLE have been described, including fatty liver, chronic persistent hepatitis, portal inflammation, hepatic vasculitis, and granulomatous hepatitis. Although these liver abnormalities may have been related to SLE, the majority of the biochemical abnormalities seen in these studies were usually secondary to other causes such as drug-induced liver dysfunction, alcoholic liver disease, congestive heart failure, infection, or metabolic disturbances.^7^ NLE liver disease may present as an isolated disorder or in association with other manifestations of NLE.^8^

The diagnosis of NLE liver disease requires that bilirubin and liver enzyme levels are consistent with cholestasis and hepatitis, along with detection in the infant of maternal antibodies to SSA/Ro and/or SSB/La. Liver biopsy is usually not indicated and should be reserved for the infants with clinical evidence of severe dysfunction or with persistent moderate dysfunction.^3^

Our patient had cutaneous lesions of NLE, with the classic ‘owl eye’ distribution. Also, CHB, cholestatic jaundice, anemia, and thrombocytopenia were present. The mother had no obvious history of systemic erythematosus lupus. The presence of anti-Ro/SSA and anti-La/SSB antibodies with high ANA titers in both the patient and the mother confirmed the diagnosis of NLE. Up to 60% of mothers of infants with NLE may be clinically asymptomatic when their children develop NLE. Also, it has been observed that the elevation of ANA titer may be an important risk marker for liver involvement in NLE,^9^ which is consistent with the findings in the present case. It is noteworthy that in the majority of reports, cholestasis and elevated liver enzymes were detected in NLE patients at around 1 month of age and later,^2^,^3^,^8^,^10^ whereas in the present case direct hyperbilirubinemia was noticed very early, i.e., about 5 days after birth.

Hematologic problems usually resolve in 2 to 3 weeks without treatment, but they may persist in some cases. There is evidence that steroids are beneficial for the patients with persistent cholestasis and pancytopenia.^3^ Our patient received prednisolone (2 mg/kg/day) for 14 days and showed improvement of thrombocytopenia. Bilirubin resolved 2 months later and liver enzymes were completely normal by 6 months.

In NLE cases, CHB is the main cause of death. Fortunately, our patient had a normal heart rate with no sign of heart failure and required no treatment. His cutaneous lupus rashes disappeared in 2 months on following the recommendations mentioned earlier.

The long-term prognosis for the children who have had NLE is still under investigation. Some cases have developed other autoimmune diseases later in childhood. In summary, NLE can present with cholestasis within a few days of birth and NLE should be considered when there is early conjugated hyperbilirubinemia, particularly if there are other associated findings.
